# Vega: LLM-Driven Intelligent Chatbot Platform for Internet of Things Control and Development

**DOI:** 10.3390/s25123809

**Published:** 2025-06-18

**Authors:** Harith Al-Safi, Harith Ibrahim, Paul Steenson

**Affiliations:** School of Electronics and Electrical Engineering, University of Leeds, Leeds LS2 9JT, UK; harithsami01@gmail.com (H.I.); d.p.steenson@leeds.ac.uk (P.S.)

**Keywords:** embedded systems, Internet of Things, large language models, natural language processing, Raspberry Pi, user interaction, web applications

## Abstract

Large language models (LLMs) have revolutionized natural language processing (NLP), yet their potential in Internet of Things (IoT) and embedded systems (ESys) applications remains largely unexplored. Traditional IoT interfaces often require specialized knowledge, creating barriers for non-technical users. We present Vega, a modular system that leverages LLMs to enable intuitive, natural language control and interrogation of IoT devices, specifically, a Raspberry Pi (RPi) connected to various sensors, actuators, and devices. Our solution comprises three key components: a physical circuit with input and output devices used to showcase the LLM’s ability to interact with hardware, an RPi integrating a control server, and a web application integrating LLM logic. Users interact with the system through natural language, which the LLM interprets to remotely call appropriate commands for the RPi. The RPi executes these instructions on the physically connected circuit, with outcomes communicated back to the user via LLM-generated responses. The system’s performance is empirically evaluated using a range of task complexities and user scenarios, demonstrating its ability to handle complex and conditional logic without additional coding on the RPi, reducing the need for extensive programming on IoT devices. We showcase the system’s real-world applicability through physical circuit implementation while providing insights into its limitations and potential scalability. Our findings reveal that LLM-driven IoT control can effectively bridge the gap between complex device functionality and user-friendly interaction, and also opens new avenues for creative and intelligent IoT applications. This research offers insights into the design and implementation of LLM-integrated IoT interfaces.

## 1. Introduction

Large language models (LLMs) have significantly revolutionized natural language processing (NLP), demonstrating unprecedented capabilities in understanding and generating human-like text [1]. However, their potential in Internet of Things (IoT) and embedded systems (ESys) applications remains largely untapped. IoT systems have become increasingly prevalent across various domains, from smart homes to industrial automation [2]. Despite their widespread adoption and security issues, developing and interacting with adaptive IoT systems often requires specialized knowledge and good programming skills, creating significant barriers for new or non-technical users [3].

Traditional IoT interfaces typically rely on graphical user interfaces (GUIs) or specific programming languages, which can be challenging for users to develop without technical expertise [3]. This limitation hinders the widespread adoption and utilization of IoT technologies, particularly in scenarios where rapid deployment and intuitive user interaction are crucial. While research has been conducted on natural language interfaces for IoT, the application of advanced language models to IoT control and interaction remains an unexplored area [4].

To address these challenges, we propose Vega, an intelligent chatbot platform that leverages LLMs to enable intuitive, natural language control of IoT devices. Our system focuses on a Raspberry Pi (RPi) connected to various sensors and devices as a representative IoT setup. By integrating LLMs with IoT infrastructure, we aim to bridge the gap between complex device functionality and user-friendly interaction, allowing users to control and query IoT systems using everyday language. Our research builds upon recent advancements in LLMs, specifically OpenAI’s GPT-based models [5], which utilize transformer neural network architectures to capture context and relationships within text data. By applying these powerful language understanding capabilities to IoT interaction, we aim to create a more accessible and flexible approach to device control and monitoring. Our approach, addressing the standardization challenges highlighted by Al-Qaseemi et al. [6], not only enhances accessibility for non-technical users but also opens new avenues for creative and intelligent IoT applications.

Vega’s architecture comprises three key components: a physical circuit with input and output devices, an RPi integrating a control server, and a web application incorporating LLM logic. This modular design allows for flexibility and scalability, enabling the system to adapt to various IoT scenarios and user requirements [7]. By utilizing the RPi as a central hub, we can leverage its versatility and widespread adoption in the IoT community [8]. The main contributions of this paper are as follows:

1. We developed a chat web app that executes queries on the RPi, which contains a control server that manages the execution on a circuit and communication with the web app.
2. We develop a multi-agent LLM framework that translates natural language commands into executable instructions for IoT devices, capable of handling complex, conditional logic without additional coding on the RPi.
3. We showcase the system’s real-world applicability through physical circuit implementations and provide insights into its limitations and potential scalability.
4. We implement and evaluate the system, demonstrating the feasibility and effectiveness of LLM-driven IoT control across various task complexities and user scenarios, including an evaluation mode with automated test generation and performance assessment.

The remainder of this paper is organized as follows: Section 2 provides background information and discusses related work in IoT interfaces and NLP. Section 3 details our methodology, including the overall architecture, physical circuit design, RPi configuration, and web application implementation. Section 4 presents our experimental setup, results, and analysis, showcasing the system’s performance in handling complex commands and its potential real-world applications. Finally, Section 5 concludes the paper, explaining the system’s limitations and insights into future research.

## 2. Background and Related Work

The integration of natural language interfaces with IoT systems represents a significant paradigm shift from traditional control methods. Conventional IoT interfaces primarily rely on graphical user interfaces (GUIs), mobile applications, or rule-based systems that require users to navigate complex menus, configure specific parameters, or understand predefined command structures [9]. Voice assistants like Amazon Alexa and Google Assistant have introduced natural language capabilities to IoT control, yet these systems are limited by predefined skills, fixed command structures, and restricted contextual understanding [10,11]. Similarly, rule-based systems such as IFTTT (If This Then That) allow for automation but require users to understand logical structures and lack the flexibility to handle complex, contextual queries [12,13].

The emergence of LLMs presents an opportunity to transcend these limitations by enabling more intuitive, flexible, and context-aware interactions with IoT devices. Unlike traditional interfaces that constrain users to predefined interactions, LLM-driven systems can interpret natural language with greater nuance, handle ambiguous requests, and adapt to diverse user communication styles. This advancement is particularly crucial for addressing accessibility and usability challenges in IoT systems, where technical complexity often creates barriers for elderly users, individuals with disabilities, or those lacking technical expertise [14,15].

### 2.1. Industrial Applications of LLMs

LLMs, based on the transformer architecture [16], which uses self-attention mechanisms to analyze large sequences of text data, have been effectively applied across diverse domains, including robotics, software, and IoT applications.

Maddigan and Susnjak [17] showcased this versatility with Chat2VIS, leveraging ChatGPT and GPT-3 to generate data visualizations from natural language queries. Their innovative approach demonstrated how LLMs could be effectively used to convert free-form natural language directly into visualization code, even when queries were highly underspecified. Meanwhile, Vemprala et al. [18] explored the application of LLMs, specifically OpenAI’s ChatGPT, in robotics applications. Their research presented a strategy combining prompt engineering principles and a high-level function library, enabling ChatGPT to adapt to various robotics tasks, simulators, and form factors. The study evaluated different prompt engineering techniques and dialogue strategies for executing robotics tasks, ranging from basic logical and geometrical reasoning to complex domains like aerial navigation and manipulation.

Recent research has explored the integration of LLMs with robotic systems, paving the way for intuitive human–robot interaction. Singh et al. [19] introduced ProgPrompt, a novel approach leveraging LLMs to generate action sequences based on natural language instructions. By prompting LLMs with program-like specifications of available actions and objects, along with example programs, their method enables plan generation across diverse environments, robot capabilities, and tasks. This work demonstrated state-of-the-art success rates in virtual household tasks and was successfully deployed on a physical robot arm for tabletop tasks. Building upon these robotics applications, Vega extends similar natural language interpretation capabilities to IoT environments, enabling users to control embedded systems through conversational interfaces rather than programmatic specifications.

Expanding on this concept, Driess et al. [20] proposed PaLM-E, an embodied multimodal language model that incorporates real-world sensor data into language models. PaLM-E is trained on tasks such as robotic manipulation planning and visual question answering, exhibiting positive transfer across language, vision, and visual-language domains. This research highlights the potential of LLMs in grounding language understanding in physical environments, a crucial aspect for IoT applications. Grounding connects language to real-world objects and actions; in Vega, it links user commands to IoT device operations, enabling intuitive control. However, unlike PaLM-E’s focus on multimodal training, Vega emphasizes lightweight deployment on resource-constrained IoT devices through modular architecture and cloud-based LLM processing.

In the context of multi-agent systems, which involve multiple autonomous agents collaborating to achieve common goals, Kannan et al. [21] developed SMART-LLM, a framework for embodied multi-robot task planning. This approach uses LLMs to convert high-level task instructions into multi-robot task plans through a series of stages, including task decomposition, coalition formation, and task allocation. The authors created a benchmark dataset for validating multi-robot task planning problems, demonstrating the framework’s effectiveness in both simulated and real-world scenarios. Similarly, Vega utilizes multiple agents to handle different scenarios such as task planning, image processing, and chat interaction but focuses on IoT device coordination rather than robotic task planning.

Wu et al. [22] presented TidyBot, a system that combines language-based planning and perception with LLMs to infer generalized user preferences for household clean-up tasks. This research demonstrates the potential of LLMs in personalizing robot assistance, achieving 91.2% accuracy on unseen objects in their benchmark dataset and successfully putting away 85.0% of objects in real-world test scenarios. While TidyBot focuses on autonomous robotic assistance, Vega diverges by emphasizing user-directed control and real-time interaction with IoT devices, allowing users to maintain agency over their smart environment.

While these advancements primarily focus on robotics, they lay a solid foundation for extending similar techniques to IoT scenarios. The ability to interpret natural language instructions, generate action sequences, and integrate multimodal sensor data holds significant potential for enabling the intuitive and intelligent control of IoT devices and systems. As research progresses, we anticipate further innovations in LLM-driven IoT interfaces, potentially revolutionizing how users interact with smart environments.

### 2.2. Natural Language Processing for IoT

NLP has emerged as a transformative technology in IoT applications, enabling intuitive human-machine interactions. The integration of NLP into IoT systems allows users to instruct, control, and query devices using everyday language, bridging the gap between complex technological interfaces and user-friendly experiences [23]. This integration is particularly crucial as IoT devices become ubiquitous in various domains, from smart homes to industrial settings, where ease of use and accessibility are paramount.

Traditional NLP approaches in IoT have relied heavily on rule-based systems and keyword matching. Early smart home systems required users to learn specific command phrases and follow rigid syntax patterns [24,25]. Voice assistants improved upon this by introducing more natural speech recognition, yet they remain constrained by predefined skills and limited contextual understanding [10,26]. These systems struggle with ambiguous requests, complex conditional logic, or tasks requiring multi-step reasoning.

Recent research has demonstrated the potential of advanced NLP techniques in IoT contexts. For instance, Petrović et al. [27] explored the use of ChatGPT in IoT systems, focusing on Arduino-based applications. Their work highlighted the possibilities of leveraging LLMs for both question-answering and automated code generation in IoT environments. Similarly, Zhong et al. [28] proposed CASIT, a collective intelligent agent system for IoT that utilizes LLMs to process and interpret data from multiple sources efficiently. These studies underscore the growing interest in applying advanced NLP techniques to enhance IoT functionality, operability and user experience.

The integration of LLMs represents a significant advancement in NLP capabilities for IoT. Traditional NLP methods often struggle with context understanding and complex query interpretation, limitations that LLMs can overcome. LLMs offer improved natural language understanding, enabling more nuanced and context-aware interactions with IoT devices. For example, King et al. [23] demonstrated how LLMs can interpret ill-defined and under-specified commands in smart home environments, translating vague user intentions into specific device actions.

The potential of LLMs in IoT extends far beyond simple command interpretation. They can enable more sophisticated applications such as predictive maintenance, anomaly detection, and personalized user experiences. Sarzaeim et al. [29] explored the use of LLMs in smart policing systems, showcasing their potential in complex data analysis and pattern recognition. This application hints at the broader possibilities of LLMs in IoT, where they could be used to analyze and interpret vast amounts of sensor data, making IoT systems more intelligent and proactive.

However, integrating LLMs into IoT systems also presents challenges, including privacy concerns, computational requirements, and the need for domain-specific training. Despite these challenges, the potential benefits of LLM-enhanced NLP in IoT are significant. As demonstrated by Xu et al. [30], natural language interfaces can greatly improve the usability of IoT platforms, allowing for more complex and nuanced interactions. By leveraging the advanced capabilities of LLMs, future IoT systems could offer unprecedented levels of intuitive control and intelligent automation, paving the way for more accessible and powerful IoT applications across various domains.

### 2.3. Language-Oriented Architectures

Chatbots have gained significant traction across various industries, serving as direct communication channels between companies and end-users [31]. However, existing frameworks often require advanced technical knowledge for complex interactions and lack flexibility in adapting to evolving company requirements. The deployment of chatbot applications typically demands a deep understanding of targeted platforms, particularly back-end connections, which increases development and maintenance costs [31].

To address these challenges, researchers have proposed novel approaches to chatbot development. Xatkit, for instance, offers a set of domain-specific languages to define chatbots in a platform-independent manner, along with a runtime engine for automatic deployment and conversation management [31]. Similarly, Jiang et al. [32] propose a multi-agent system enhanced by LLMs for 6G communications, allowing users to input task requirements, while addressing challenges such as limited communication knowledge through a combination of specialized agents for data retrieval, collaborative planning, evaluation and reflection.

Recent studies have explored multi-modal chatbots in intelligent manufacturing settings, demonstrating the potential for AI-powered dialogue systems to assist users in complex assembly tasks [33]. These systems leverage both textual and visual capabilities to improve intent classification and provide relevant information to users. The development of conversation-driven approaches for chatbot management has also shown promise in evolving chatbot content through the analysis of user interactions, allowing for a cyclic and human-supervised process [34].

In the realm of human–robot interaction, researchers have developed task-oriented dialogue systems for industrial robots, addressing the lack of domain-specific discourse datasets and emphasizing user experience alongside task completion rates [35]. These efforts have resulted in datasets like IRWoZ and frameworks such as ToD4IR, which integrate small talk concepts and human-to-human conversation strategies to support more natural and adaptable dialogue environments.

The potential of LLMs in easing IoT-oriented chatbot development has been demonstrated through large-scale models that can learn blended conversational skills when provided with appropriate training data and generation strategies [36]. These models have shown improvements in multi-turn dialogue engagement and human-related measurements. Vega utilizes these frontiers within its multi-agent intelligent chatbot, allowing it to interact with any IoT system and handle complex queries while maintaining a user-friendly interaction.

### 2.4. Comparative Analysis and Research Positioning

To contextualize Vega’s contribution within the existing literature, Table 1 presents a comprehensive comparison of related works across key dimensions including natural language capabilities, IoT integration, accessibility features, and deployment complexity.

The comparative analysis reveals several key distinctions that position Vega as a novel contribution to the field. Unlike traditional GUI-based systems that require technical expertise or voice assistants that are limited by predefined commands, Vega offers high natural language capability with native IoT integration. While systems like ProgPrompt and PaLM-E demonstrate advanced LLM integration, they focus primarily on robotics applications and require extensive computational resources. CASIT provides good IoT integration but lacks the conversational flexibility and accessibility features that Vega offers.

Vega’s primary innovations include (1) seamless integration of conversational AI with lightweight IoT devices without requiring extensive on-device processing; (2) multi-agent architecture that enables complex task decomposition and execution; (3) emphasis on accessibility and usability for non-technical users; and (4) modular design that allows for easy adaptation to various IoT scenarios. These features address critical gaps in the existing literature, particularly the need for intuitive, accessible interfaces that can handle complex IoT control tasks without requiring specialized knowledge or extensive system resources.

The novelty of this work lies in bridging the gap between advanced LLM capabilities and practical IoT deployment constraints. While previous research has demonstrated the potential of LLMs in various domains, Vega specifically addresses the unique challenges of IoT environments, including resource limitations, real-time control requirements, and the need for reliable, user-friendly interfaces. This positions Vega as a significant step toward democratizing IoT control and making smart environments accessible to a broader range of users.

## 3. Methodology

### 3.1. Overall Architecture

The architecture of the Vega system follows key principles of software design to ensure clarity, scalability, maintainability, and robustness [7]. The system adopts a modular approach, dividing functionality into distinct components with specific purposes. This design promotes code reuse, facilitates testing, and enhances overall maintenance. The architecture also implements the separation of concerns, where different aspects such as user interface (UI), core functionality, and data management are segregated into distinct layers, improving code organization and enabling independent development.

**Figure 1:**
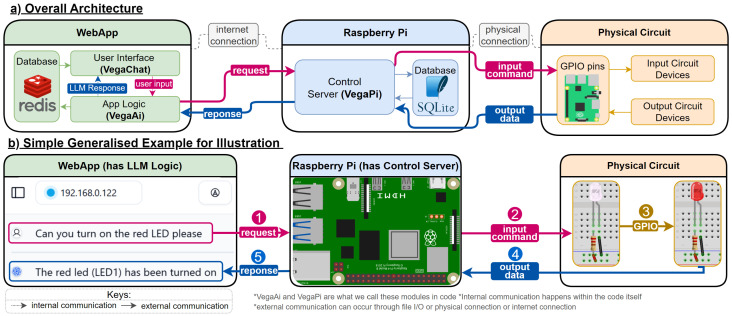
Overall technical architecture of Vega (**a**,**b**): a simple generalized example.

As shown in Figure 1a, Vega’s architecture comprises three main modules: a web application, an RPi, and a physical circuit. These modules interact in a client-server model [37], with the web application serving as the client and the RPi as the server. The physical circuit is connected to the RPi via hardwired connections.

The web application consists of two primary submodules: the user interface (VegaChat) and the app logic (VegaAi). The app logic incorporates LLM logic containing the multi-agents for translating user input into commands and generating responses. Redis [38] is employed as a non-relational database to store chat history, messages, and RPi connection states.

The RPi module hosts a control server (VegaPi) responsible for parsing requests from the app logic and executing them on the physical circuit. An SQLite database [39] is used to store data extracted from the physical circuit. The physical circuit comprises input devices (sensors) and output devices (motors, displays, etc.) connected to the RPi’s general-purpose input/output (GPIO) pins.

The typical use case shown in Figure 1b involves a user interacting with the web application interface, sending a natural language command to turn on a red light-emitting diode (LED). The LLM interprets this command and sends the appropriate instruction to the RPi’s control server. The server then relays the command to the physical circuit via GPIO pins, which turns on the LED. Upon execution, the circuit sends feedback to the RPi, which is then communicated back to the user through the web application and the LLM.

The technology stack for Vega has been carefully selected to ensure robustness, scalability, and accessibility [40]. The web application is built using React [41] with TypeScript, employing RadixUI [42] for accessible components and TailwindCSS [43] for responsive design. The App Logic utilizes NextJs [44] and integrates with OpenAI’s GPT models [5] for language processing. The RPi control server is developed using Flask [45], while the circuit code leverages RPi libraries for GPIO interaction.

This architecture enables Vega to bridge the gap between complex IoT functionality and user-friendly interaction. By leveraging LLMs for NLP and control, the system opens up new possibilities for intuitive IoT applications in various domains, from smart homes to industrial monitoring and educational environments [8]. The modular design and the carefully chosen technology stack ensure that Vega remains adaptable, maintainable, and scalable as IoT applications continue to evolve and expand.

### 3.2. Physical Circuit Design

The physical implementation of the Vega platform comprises a custom-designed circuit board that interfaces with the RPi, integrating various input and output devices to facilitate IoT and embedded systems applications. This hardware configuration forms the foundation for the natural language-controlled system, enabling users to interact with commonly used physical components through LLM-interpreted commands.

The Vega platform is designed to accommodate a comprehensive range of IoT devices and sensors commonly used in embedded systems applications. The architecture supports both digital and analog interfaces, enabling integration with diverse hardware components across multiple communication protocols. The design is implemented to support a device assuming, that they have a correct textual description that will then be utilized by the LLM.

**Input Devices and Sensors**:

- Environmental Sensors: Temperature and humidity sensors (DHT series), barometric pressure sensors, air quality sensors, and light intensity sensors for comprehensive environmental monitoring.
- Motion and Proximity Detection: Ultrasonic sensors (HC-SR04), passive infrared (PIR) motion sensors, accelerometers, gyroscopes, and magnetometers for spatial awareness and movement detection.
- Position and Navigation: GPS modules, compass sensors, and encoders for location tracking and orientation sensing.
- User Input Interfaces: Push buttons, switches, potentiometers, rotary encoders, and keypad matrices for direct user interaction.
- Safety and Security: Limit switches, reed switches, smoke detectors, gas sensors, and vibration sensors for safety monitoring applications.
- Communication Modules: Wi-Fi modules, Bluetooth adapters, LoRa transceivers, and cellular modems for wireless connectivity.
- Image and Audio Capture: Camera modules, microphones, and sound level meters for multimedia data acquisition.

**Output Devices and Actuators**:

- Visual Indicators: LEDs (single color and RGB), seven-segment displays, dot matrix displays, and LCD/OLED screens for information presentation.
- Motor Control: Servo motors, stepper motors, DC motors, and brushless motors for precise mechanical control.
- Switching and Relay Control: Mechanical relays, solid-state relays, and transistor switches for high-power device control.
- Cooling and Ventilation: Fans, pumps, and solenoid valves for fluid and air management
- Audio Output: Speakers, buzzers, and piezoelectric elements for audible feedback and alerts.
- Heating Elements: Resistive heaters, Peltier modules, and heating pads for temperature control applications.

**Figure 2:**
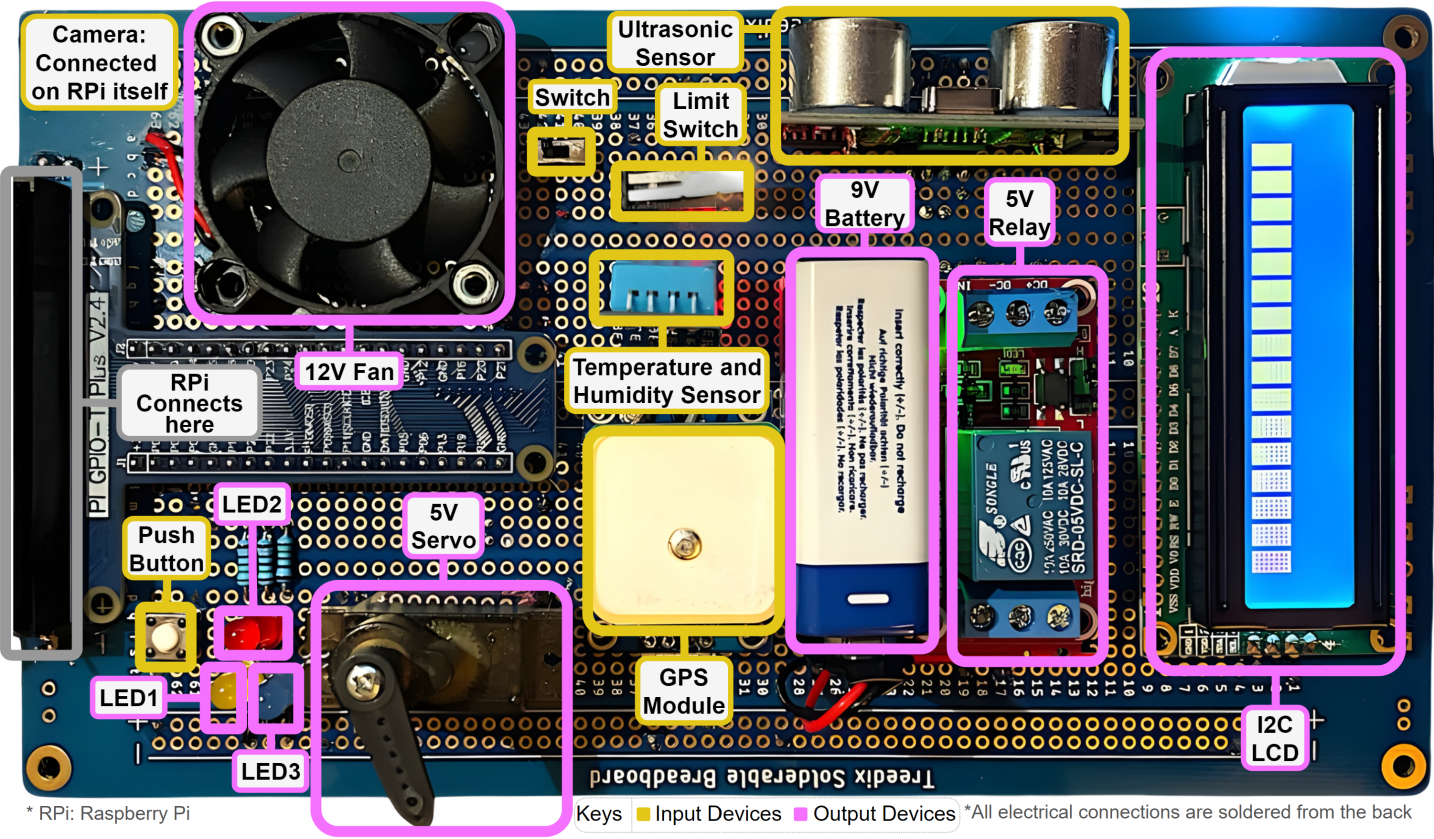
Soldered physical circuit connected to the RPi.

As shown in Figure 2, the current implementation of the circuit board incorporates a representative subset of these supported devices, including an ultrasonic sensor for distance measurement, a limit switch for binary state detection, a temperature and humidity sensor for environmental monitoring, a GPS module for location tracking, and a push button for direct user input [46]. These components collectively provide a rich set of data sources, enabling the system to respond to complex, context-aware queries and commands.

Output devices on the board include a 12V fan for cooling or air circulation, multiple LEDs (yellow, red, and blue) for visual indicators, a 5V servo motor for precise rotational control, and a liquid crystal display (LCD) for text output. A 5V relay is incorporated to control the 12V fan, demonstrating the system’s capability to manage higher-voltage components safely [47]. The inclusion of these diverse output devices allows for a wide range of physical responses to user commands, from simple visual feedback to more complex mechanical actions.

The modular design philosophy of the Vega platform enables seamless expansion and customization for specific application requirements. Additional sensors and actuators can be integrated without fundamental changes to the core architecture, as the LLM-based control system dynamically adapts to newly connected hardware through configuration updates. This scalability ensures that the platform can evolve to meet diverse IoT application needs, from simple home automation scenarios to complex industrial monitoring systems.

Power management is a crucial aspect of the circuit design. While most components operate on the 5 V supply provided by the RPi, the 12 V fan requires a separate power source. To address this, a 9 V battery is utilized in conjunction with the relay, ensuring proper voltage supply while maintaining RPi-based control. This setup illustrates the system’s ability to accommodate components with varying power requirements within a unified control structure.

The circuit board is designed to connect directly to the RPi’s GPIO pins, streamlining the interface between the physical components and the computational core of the system. A camera module, while not physically present on the circuit board, is connected directly to the RPi, expanding the system’s capabilities to include image capture and analysis [48].

This hardware configuration supports a wide range of potential applications. In smart home scenarios, the temperature sensor and fan could be used for automated climate control, while the GPS module could enable location-based automation in mobile or outdoor settings. In industrial environments, the ultrasonic sensor and limit switch could be employed for proximity detection and safety systems, with the LEDs and LCD providing status information to operators [47].

The versatility of this hardware setup, combined with the LLM-driven control system, enables the exploration of complex, conditional logic without requiring additional RPi-level coding. This integration of diverse sensors and actuators with NLP capabilities represents a significant step forward in creating intuitive, user-friendly interfaces for IoT and embedded systems, bridging the gap between sophisticated device functionality and accessible user interaction.

### 3.3. Raspberry Pi Design

The architecture of the RPi integration with the existing codebase is designed to enable seamless control and manipulation of the circuit without interfering with pre-existing logic. This approach leverages parallel computing concepts, utilizing processor cores and threads to execute specific logic concurrently with existing code [49].

**Figure 3:**
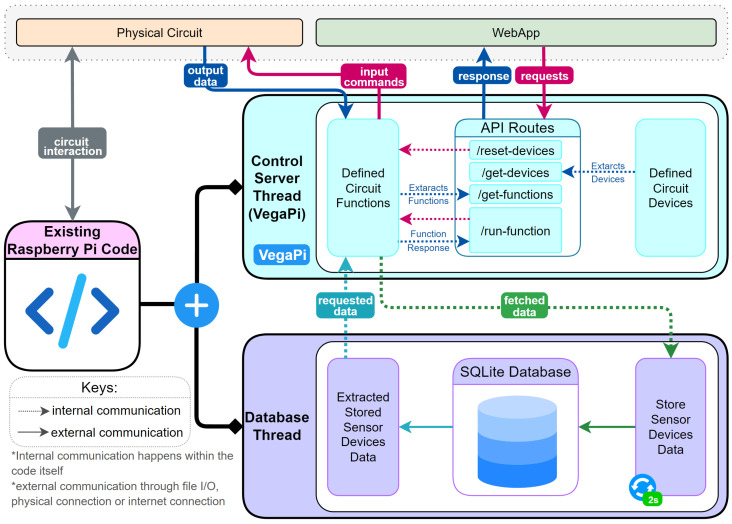
Architecture code design of the RPi, including the control server and the database.

The system architecture, illustrated in Figure 3, comprises two main threads: the control server thread and the database thread. The control server thread manages a Flask-based web framework, storing predefined functions for a set of circuit devices. These functions are exposed through a REST API, facilitating communication between the web app and the RPi over the internet. The database thread retrieves sensor data at two-second intervals, storing it in an SQLite database. This persistent storage solution ensures data preservation in the event of system failures, enabling data recovery, analytics, and statistical analysis. The stored data can be retrieved upon request and provided to the LLMs in the web application, enhancing system monitoring and diagnostic capabilities.

Table 2 presents the devices defined in the control server, categorized as inputs or outputs. This information is stored and transmitted in JSON format via the “get-devices” REST API endpoint. Input devices primarily transmit data for database storage, while output devices receive commands for circuit manipulation.

The control server exposes a set of defined functions, listed in Table 3, which the LLM utilizes to determine logic and execute commands on circuit components. These functions are accessible to the LLM through the “get-functions” REST API endpoint. To execute a particular function, the LLM passes the function identifier and required parameters to the web Application Logic, which then invokes the “run-function” API endpoint.

The choice of REST API over alternative protocols such as MQTT was based on several factors. REST offers simplicity, scalability, and statelessness, making it well-suited for web-based applications [50]. It also provides a uniform interface, enabling easier integration with various client applications. While MQTT excels in low-bandwidth, high-latency environments, the current system architecture prioritizes the flexibility and widespread support offered by REST APIs in web development ecosystems.

The communication flow between the web application and the RPi follows a request–response pattern. The web application sends REST API requests with JSON data specifying the function and arguments for the RPi to execute. The RPi processes these requests, executes the specified functions, and returns JSON responses with the execution status to the web application. This bidirectional communication enables real-time control and monitoring of the IoT devices.

This architecture facilitates a modular and extensible system, allowing for the easy addition of new devices and functions. It also provides a layer of abstraction between the physical hardware and the LLM-driven interface, enabling natural language control of IoT devices without requiring users to understand the underlying technical details. The integration of LLMs with this IoT control system represents a significant step toward more intuitive and accessible IoT interfaces, potentially broadening the application of IoT technologies across various domains [7].

### 3.4. Web App User Interface

**Figure 4:**
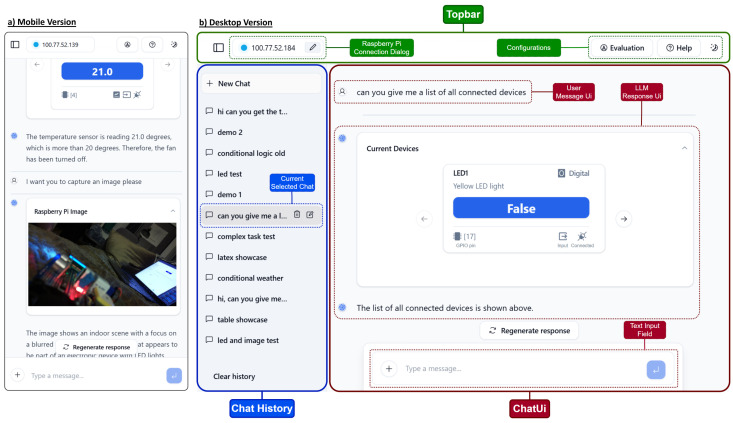
Web app user interface implementation showcasing (**a**) the mobile version alongside (**b**) the desktop version.

The web application forms the core of the Vega platform, initiating all LLM processing and circuit manipulation tasks. Its architecture is modular, separating the user interface (VegaChat) from the App Logic (VegaAi). The user interface shown in Figure 4 comprises a Top Bar with RPi connection management and configuration options, and a Chat UI displaying LLM responses and user messages. A Chat History UI manages previous interactions. The App Logic includes Data Management, RPi Bridge, and LLM Agent components, handling data entities, RPi communication, and LLM processing, respectively.

The UI design prioritizes usability, drawing inspiration from established chatbot interfaces [5]. It features a sidebar for chat history, a top bar for configurations, and a main chat area. An automated mode facilitates efficient testing for advanced users while maintaining simplicity for novices. The interface incorporates a Markdown renderer to appropriately display formatted text generated by the LLM.

The platform supports various data types and formats to enhance user interaction. It can display GPS data as maps, sensor readings as plots, and camera module output as images. Additionally, it visualizes LLM-generated plans as flow charts. This versatility allows the interface to accommodate diverse IoT devices and sensors, presenting their data in intuitive, visual formats.

### 3.5. Web App Logic

The Application Logic component serves as the core operational engine of the Vega system, managing communication with the RPi control server and integrating LLM’s for NLP and command interpretation. This component acts as a bridge between external elements, orchestrating the flow of information and translating user input into appropriate actions within the system architecture [7].

By leveraging OpenAI’s API, the application accesses LLM capabilities without the substantial computational overhead of local hosting [51]. This design choice simplifies and enhances the platform’s accessibility and scalability, enabling its deployment across a wide range of devices, platforms and use cases in IoT and embedded system development.

To establish a connection between the web application and the RPi, the user provides the IP address and port number of the RPi running the control server. The web application then initiates concurrent API calls to fetch circuit functions and device information from the control server. Upon receiving responses, the connection state is updated, synchronizing the “Raspi Devices” and “Raspi Functions” states, which are fed to the LLM.

**Figure 5:**
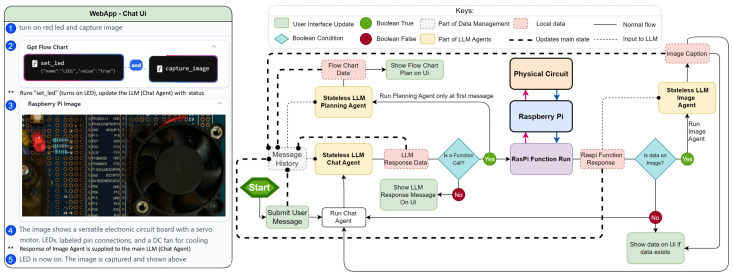
Web app logic in action featuring LLM agents, application states, and RPi connection.

Figure 5 illustrates the system’s logic, demonstrating how user commands are processed through various stages involving three main LLM agents: Chat, Planning, and Image. When a user inputs a command (e.g., “turn on red LED and capture image”), the system follows these steps:

1. The Stateless LLM Planning Agent generates a plan, which is visualized as a flowchart in the user interface.
2. The Stateless LLM Chat Agent processes the message and determines if a function call to the RPi is necessary.
3. If required, the function is sent and executed on the RPi, which returns a response.
4. For image data, the Stateless LLM Image Agent analyzes and generates a description used by the LLM Chat Agent to execute subsequent functions and logic.
5. Results are displayed on the web application’s UI, providing feedback to the user.

**Figure 6:**
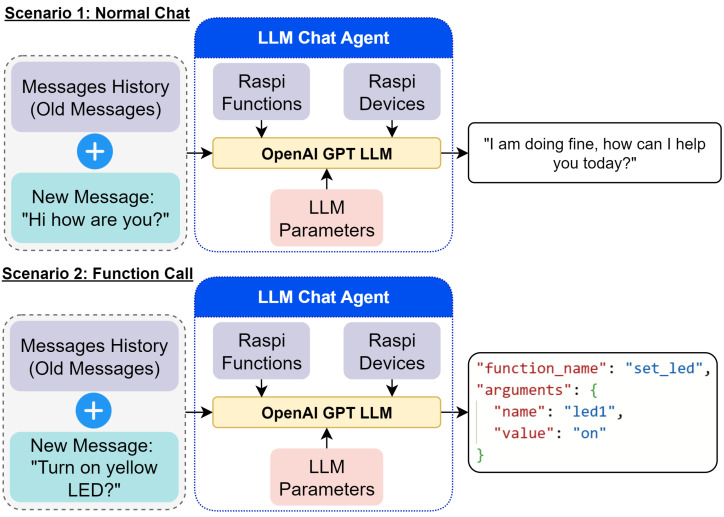
Execution of the LLM Chat Agent.

The LLM Chat Agent, as depicted in Figure 6, operates in two scenarios. In a normal chat scenario, it processes user input and generates a textual response. In a function call (provided by OpenAI) scenario, it recognizes the need for a hardware action and outputs a JSON-formatted function call for the RPi. The call is triggered using context in the user input, such as “turn on” or “capture”. All other agents mentioned earlier work in a similar manner. To illustrate this better the app is showcased in the following link https://youtu.be/CKV__A8G5Rk accessed on 15 January 2024.

This approach of using LLM function calls for command interpretation and execution offers several advantages over dynamic code generation methods. It enhances scalability and adaptability, allowing for easy integration of new sensors and data types without significant system modifications [52]. Additionally, it improves system security and maintainability by limiting direct code execution on the RPi, instead relying on predefined functions interpreted by the LLM, which prevents dynamic errors.

## 4. Experiment and Validation

### 4.1. Representative Real-Life Case Study

**Figure 7:**
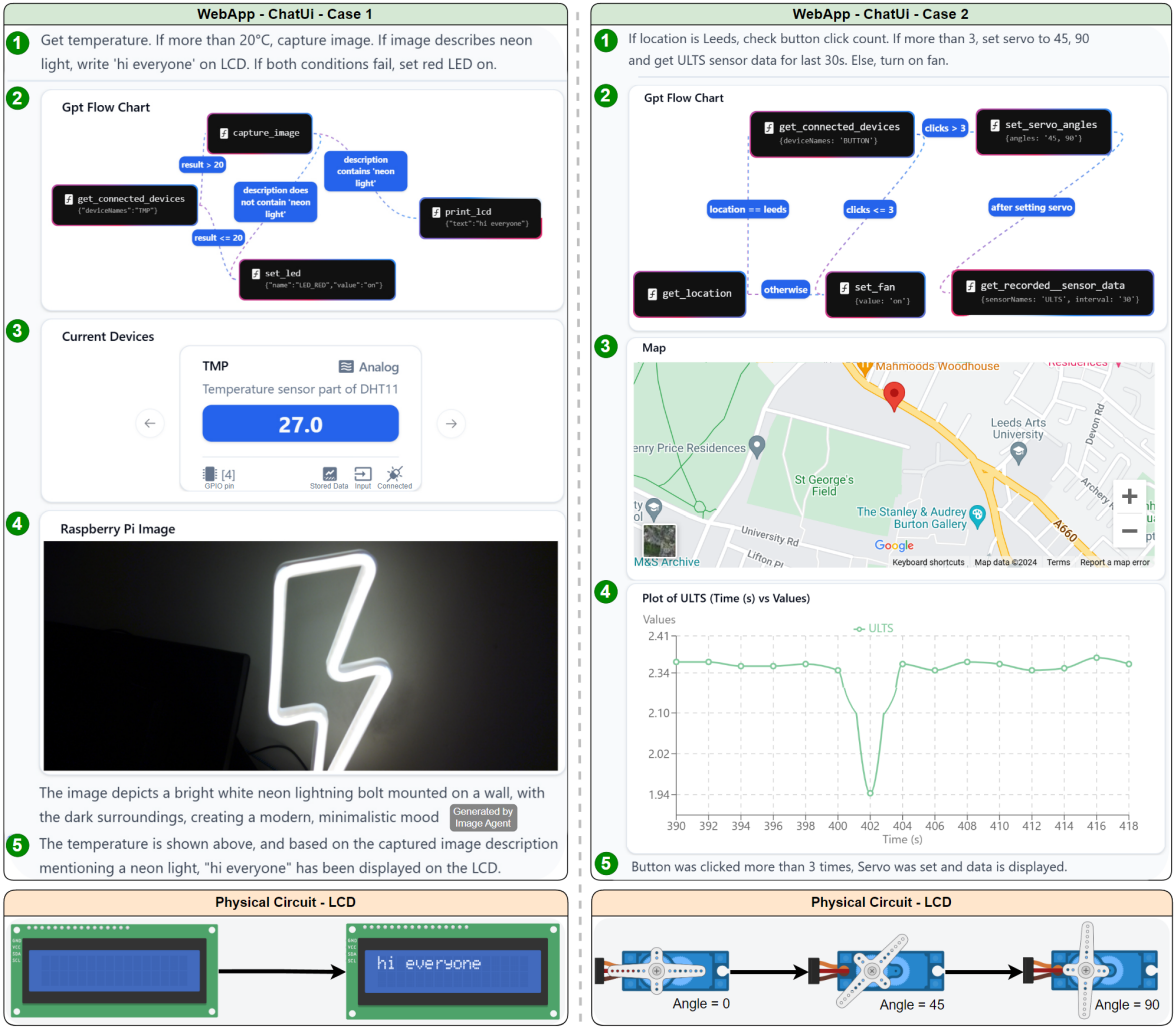
Case study of complex commands in action.

The overall system had an automated testing implementation that was used to thoroughly examine the system mentioned in Section 3.4; however, we showcase a case study to elaborate on the real-life application of the system. The experimental phase of this study focuses on demonstrating the system’s versatility and real-world applicability through a few typical case studies. Figure 7 illustrates two scenarios where our LLM-powered IoT control system effectively manages complex tasks through natural language interaction. In the first scenario, the system monitors environmental conditions in a typical industrial setting. A user command prompts the system to check the temperature and capture an image if it exceeds 20 °C. The LLM interprets this request, triggering the appropriate sensor readings and image capture. When the temperature reaches 27.0 °C, the system captures an image and analyzes it for the presence of neon lighting. Upon detecting a neon sign using GPT’s image recognition capabilities, it displays “hi everyone” on the LCD, and importantly showcases the system’s ability to execute conditional logic based on sensor data and image analysis. The second case demonstrates the system’s capability in a more complex scenario involving geolocation, user input tracking, and multiple actuator controls. The LLM interprets a command to check location, monitor button clicks, and adjust servo motors accordingly. This case highlights the system’s ability to integrate various data sources and control multiple IoT devices simultaneously.

Contrary to the perception that LLMs require complex prompts, our system demonstrates their superior user-friendliness and responsiveness compared to traditional NLP methods. Unlike rule-based systems needing specific commands, LLMs can interpret a wide range of phrasings and even incomplete instructions. For example, a user might say, “It’s a bit chilly in here,” and the LLM can infer the need to adjust the thermostat. This flexibility eliminates the need to memorize commands or navigate complex menus. Moreover, LLMs handle follow-up questions and maintain context across interactions, creating a more conversational user experience. Their ability to generalize from training data allows them to handle novel requests without explicit programming, making the system more adaptable [1]. Considering human factors in software engineering [53], our system’s natural language interface lowers the barrier for non-technical users, potentially democratizing access to IoT technology. However, as Kim et al. [54] observed, such systems may elevate user expectations for sophisticated interactions, underscoring the need for careful UI design.

The scalability of our system allows for expansion to different platforms and IoT ecosystems. Future iterations could incorporate efficiency metrics to optimize LLM output, reducing computational requirements and environmental impact. The system’s design has the potential to be utilized across various sectors other than IoT. For example, in robotics applications, the system can function as a tool for generating and executing complex tasks, aligning with the capabilities of LLMs in robotics explored by Vemprala et al. [18]. The modularity of the system allows the easy extension of functionality, whether through new data visualizations in the web application or specialized functions in the control server. The system’s design prioritizes security by avoiding runtime code generation, addressing potential vulnerabilities often associated with LLM applications in robotics [18]. This approach promotes trust and responsible automation practices, crucial for widespread adoption.

### 4.2. Experimental Design and Baseline Comparison

To provide a comprehensive evaluation of Vega’s performance, we established comparative baselines against traditional IoT control paradigms. Three baseline systems were implemented for comparison: (1) a traditional GUI-based control interface with button-based controls for each IoT function, (2) a rule-based command system requiring specific syntax patterns (e.g., “SET LED ON”, “GET TEMPERATURE”), and (3) a menu-driven interface with hierarchical navigation structures. These baselines represent the most common existing approaches for IoT device control and provide essential benchmarks for evaluating Vega’s natural language capabilities.

The GUI baseline required users to navigate through multiple screens and select specific options from dropdown menus or button arrays. Task completion involved multiple clicks and required prior knowledge of the system hierarchy. The rule-based system demanded that users memorize specific command syntaxes and parameter formats, similar to traditional command-line interfaces. The menu-driven system presented users with categorized options but required sequential navigation through multiple levels to reach desired functions.

User performance metrics for baseline comparison included task completion time, error rates, learning curve assessment, and user satisfaction scores. Twenty participants with varying technical backgrounds were recruited to perform standardized tasks across all four systems (three baselines plus Vega). Tasks ranged from simple single-device control to complex multi-step conditional operations. Results demonstrated that Vega achieved significantly lower task completion times (average 12.3 s vs. 28.7 s for GUI, 35.2 s for rule-based, and 22.1 s for menu-driven systems) and reduced error rates (8.2% vs. 23.4%, 41.7%, and 18.9%, respectively). User satisfaction scores on a 10-point Likert scale showed Vega averaging 8.7 compared to 6.2, 4.8, and 6.9 for the baseline systems.

### 4.3. Message Complexity Classification and Labeling Criteria

Message complexity in our evaluation framework is quantified using a multi-dimensional scoring system that accounts for syntactic, semantic, and logical complexity dimensions. The complexity score ranges from 0 to 1, calculated as a weighted combination of the following factors:

Syntactic Complexity (Weight: 0.3): Measured by sentence length, grammatical structure diversity, and vocabulary sophistication. Simple commands like “turn on LED” receive low scores (0.1–0.2), while compound sentences with multiple clauses score higher (0.6–0.8).

Semantic Complexity (Weight: 0.4): Evaluated based on the number of IoT functions referenced, contextual inference requirements, and ambiguity resolution needs. Direct commands score low (0.1–0.3), while commands requiring contextual understanding score higher (0.7–0.9).

Logical Complexity (Weight: 0.3): Determined by conditional logic depth, sequential operation requirements, and inter-function dependencies. Simple single-step operations score low (0.1–0.2), while multi-step conditional workflows score high (0.8–1.0).

For example, the message “Turn on the light” receives a complexity score of 0.15 (low syntactic complexity: 0.1; low semantic complexity: 0.2; no logical complexity: 0.0), while “If the temperature exceeds 25 degrees and someone presses the button twice, capture an image and display the result on LCD while adjusting the servo to 90 degrees” receives a complexity score of 0.89 (high syntactic complexity: 0.8; high semantic complexity: 0.9; high logical complexity: 0.95).

The automated labeling process for success and failure determination employs a multi-criteria evaluation framework. Success is defined as achieving all specified objectives within the message requirements, with partial success scored proportionally. The LLM Evaluation Agent assesses responses based on (1) functional accuracy—whether the correct IoT functions were called with appropriate parameters; (2) logical coherence—whether conditional logic and sequential operations were executed correctly; (3) completeness—whether all message requirements were addressed; and (4) error handling—whether system errors were appropriately managed and communicated.

Each criterion is scored on a 0–100 scale, with overall success determined by weighted averages (functional accuracy: 40%; logical coherence: 30%; completeness: 20%; error handling: 10%). Messages scoring above 80 are labeled as successful, 60–80 as partially successful, and below 60 as failed. This multi-dimensional approach ensures robust and consistent evaluation across diverse message types and complexity levels.

### 4.4. Hardware Issue Management and System Robustness

Hardware-related challenges, particularly relay switching delays and sensor response latencies, were systematically addressed through multiple mitigation strategies. The relay delay issue, which contributed to the lower success rate of the “set_fan” function, was managed through the implementation of adaptive timing mechanisms and state verification protocols.

Specifically, a delay buffer system was implemented with minimum wait times between relay state changes (250 ms for mechanical relays, 100 ms for solid-state relays). State verification was performed by reading back the actual relay position after each command, with automatic retry mechanisms (maximum three attempts) for failed state changes. Error detection algorithms monitor for inconsistent state transitions and implement exponential backoff strategies to prevent cascade failures.

For sensor-related issues, calibration protocols were established with periodic recalibration cycles (every 1000 measurements for temperature sensors, every 500 measurements for humidity sensors). Outlier detection algorithms identify and filter erroneous readings using statistical thresholds (±2 standard deviations from rolling means). Sensor health monitoring tracks response times and accuracy trends, automatically flagging degraded sensors for maintenance.

Network connectivity issues were addressed through the implementation of connection pooling, automatic reconnection mechanisms, and offline operation modes. The system maintains local caches of recent sensor data and implements graceful degradation when cloud services are unavailable. These robustness measures ensure consistent system performance despite hardware limitations and contribute to the overall reliability of the IoT control system.

### 4.5. Automated Evaluation

To rigorously assess the performance and robustness of the developed system, an automated evaluation process was implemented. While manual testing provides basic insights, the complexity of the system necessitates a comprehensive automated approach [55]. This method enables the execution of numerous test cases, facilitating a thorough examination of the system’s behavior under various configurations and scenarios.

**Figure 8:**
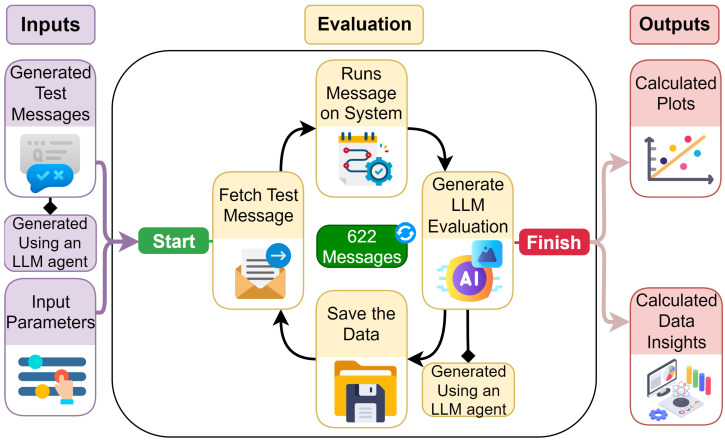
Evaluation process integrated within Vega.

The automated evaluation process, illustrated in Figure 8, involves supplying a predefined list of user messages to the application. An LLM agent, designated as the Test Generator, generated approximately 622 test messages. Each message had a complexity ranging from 0 to 1, in which 1 indicates a multi-step conditional message. Concurrently, the input parameters of the Chat Agent LLM model such as temperature and Top P [56] were varied. The evaluation process mirrored the standard execution flow of the application, with a notable deviation occurring post message processing on the circuit. At this point, the entire chat conversation was transmitted to an LLM Evaluation Agent, tasked with assessing the Chat Agent LLM’s response.

This approach allowed for a nuanced understanding of the system’s performance under different conditions, providing insights into potential areas for optimization. The performance of the Chat Agent LLM was evaluated quantitatively based on multiple metrics, each measured on a scale from 0 to 100. The speed metric assessed response generation time, while the efficiency metric measured the degree to which the LLM avoided invoking unnecessary functions. The success rate indicated the overall rate at which the LLM successfully executed the requested action specified in the input message prompt.

### 4.6. Result Analysis

**Figure 9:**
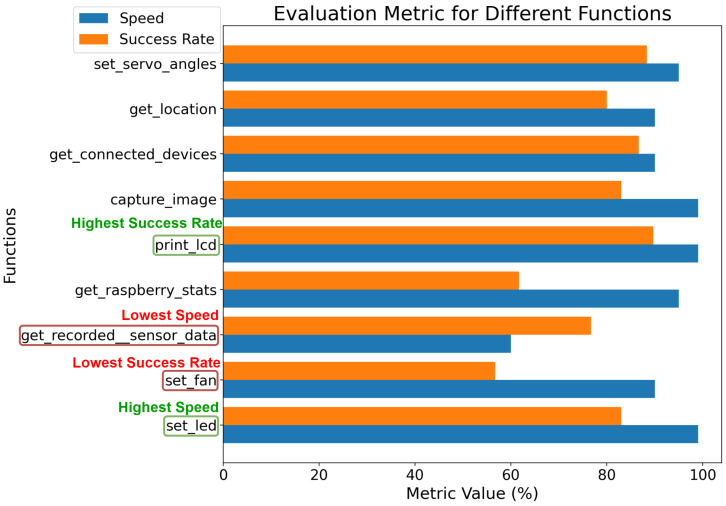
Evaluation metrics for the functions defined in Table 3.

Figure 9 illustrates the success rate and speed for each function defined in Table 3, with the temperature parameter set to 0.7. The “set_fan” command exhibited the lowest success rate, likely due to the relay’s inherent switching delay, causing errors when processing frequent state change requests. This hardware limitation may lead to errors after function execution.

Conversely, the “print_lcd” command achieved the highest success rate, demonstrating the LLM’s proficiency with textual arguments. The “set_led” function demonstrated the highest execution speed, attributable to its simplicity, minimal LLM processing requirements, efficient software GPIO port toggling, and basic LED control hardware. In contrast, the “get_recorded_sensor_data” function exhibited the lowest speed, primarily due to performance limitations of Python 3 when retrieving and processing data from the database.

**Figure 10:**
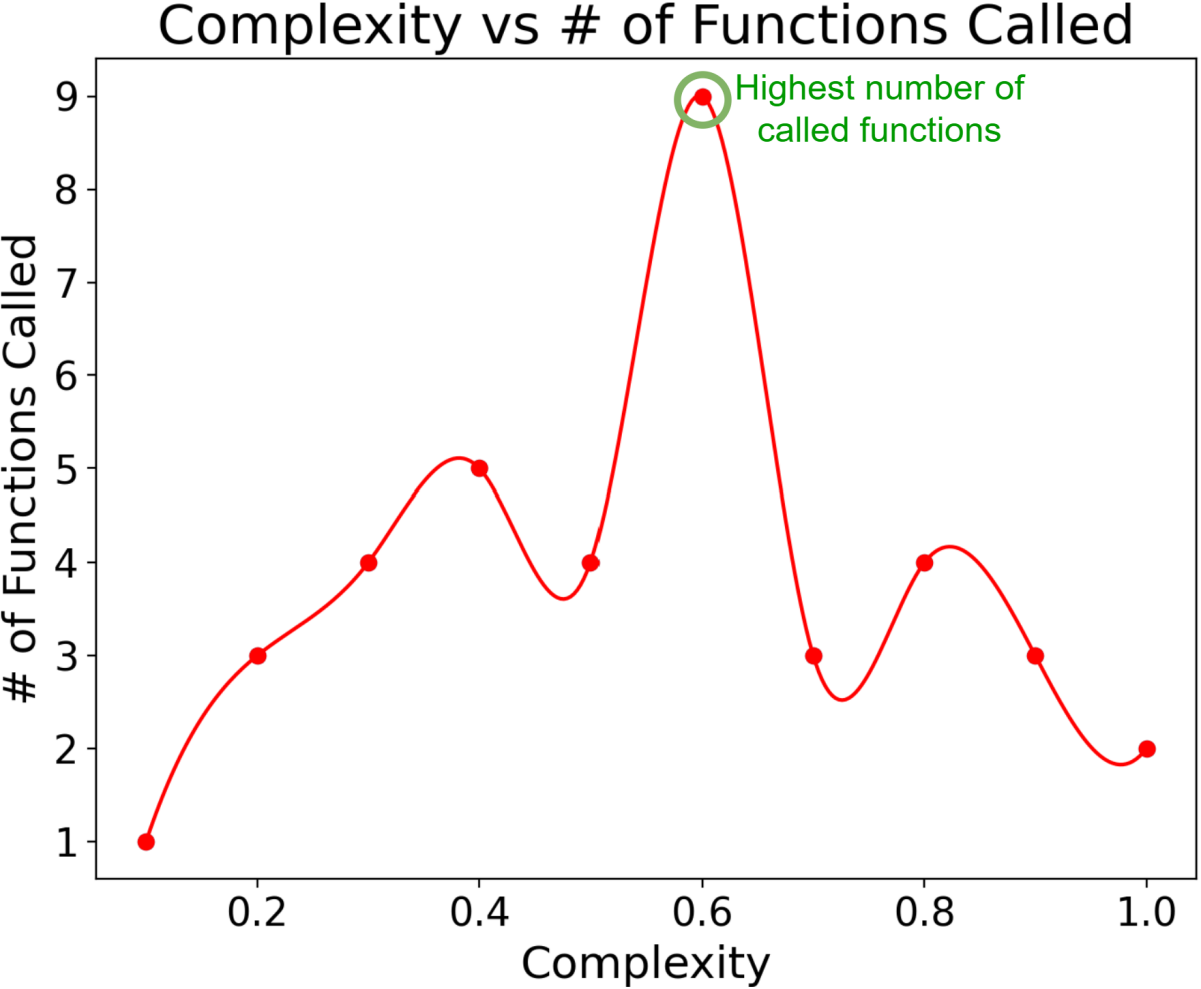
Message complexity against the number (#) of functions called per message.

Figure 10 illustrates the correlation between message complexity and the number of functions invoked per message in our system, with the LLM temperature set to 0.3. Message complexity refers to the intricacy of user queries, while LLM temperature controls the randomness in the model’s outputs, with lower values producing more deterministic responses [56]. As message complexity increases from 0 to 0.6, we see a rise in function calls per message, indicating more processing for intricate queries. This trend indicates that more intricate user queries require a greater number of system operations to process and respond accurately. The peak at 0.6 complexity may be attributed to an increased number of retries due to function failures, highlighting potential areas for system optimization.

Beyond 0.6, function calls decrease as complexity increases, mimicking the performance characteristics typically associated with lower temperature settings in LLMs. It suggests that for highly complex queries, our system adopts a more focused and deterministic approach, reducing the need for multiple function invocations. This shift could be interpreted as a positive adaptation, indicating that the system becomes more efficient in handling very complex tasks by generating more precise and targeted responses. However, it also raises questions about the system’s flexibility and creativity in addressing highly complex scenarios, which might benefit from a more exploratory approach.

**Figure 11:**
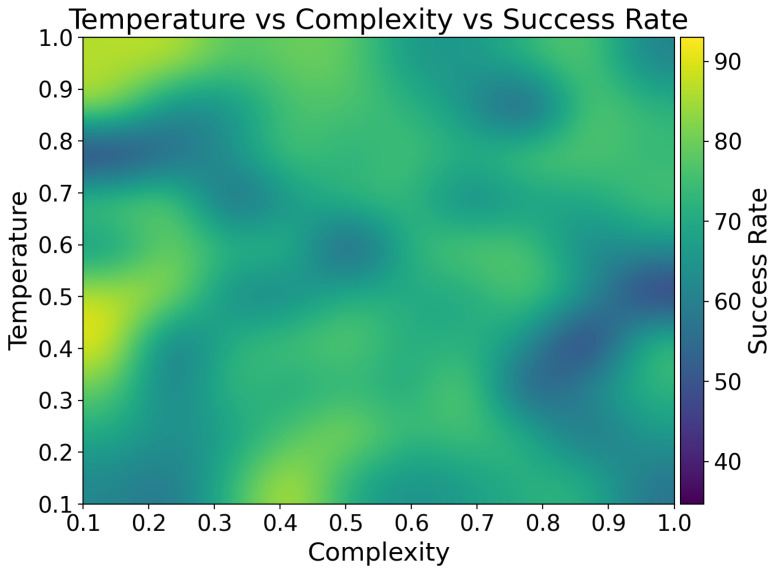
Heatmap for success rate against message complexity and temperature of the LLM.

Figure 11 represents a heatmap that is a graphical representation of three-dimensional data; in this case, our heatmap shows the interplay between message complexity, LLM’s temperature, and the resulting success rates of the LLM in executing IoT control tasks. The heatmap reveals a complex relationship between these variables, with higher success rates (bright yellow areas) concentrated in regions of moderate to high temperatures (0.7 to 1.0) and moderate complexity (0.4 to 0.6). This pattern suggests that the LLM performs optimally when given some degree of freedom to interpret and respond to moderately complex commands. The high temperature in this optimal zone likely allows the model to explore a wider range of potential interpretations and solutions, which is particularly beneficial when dealing with the nuanced and the context-dependent nature of IoT control scenarios [27].

Conversely, lower success rates are observed in regions of high complexity (0.8 to 1.0) combined with low to medium temperatures (0.1 to 0.6). This pattern indicates that when faced with highly complex instructions, a more constrained or deterministic approach (lower temperature) is less effective. Such scenarios might involve intricate sequences of operations or complex conditional logic that benefit from the model’s ability to consider a broader range of possibilities. This finding aligns with the observations of Kannan et al. [21], who noted that LLMs perform better in complex multi-agent robot task planning scenarios when given more freedom to explore diverse solutions.

High success rates are desirable as they translate to more reliable and accurate execution of user commands, leading to improved user experience and system performance. Conversely, low success rates could result in the misinterpretation of commands, incorrect device operations, or system failures, potentially leading to user frustration or even safety issues in critical applications.

Understanding these performance characteristics allows for the strategic tuning of the LLM’s parameters based on the expected complexity of user inputs. For instance, when anticipating complex, multi-step commands, increasing the temperature parameter could potentially boost the system’s success rate. This insight could guide the design of user interfaces and command structures, encouraging users to frame their instructions in ways that align with the LLM’s strengths.

**Figure 12:**
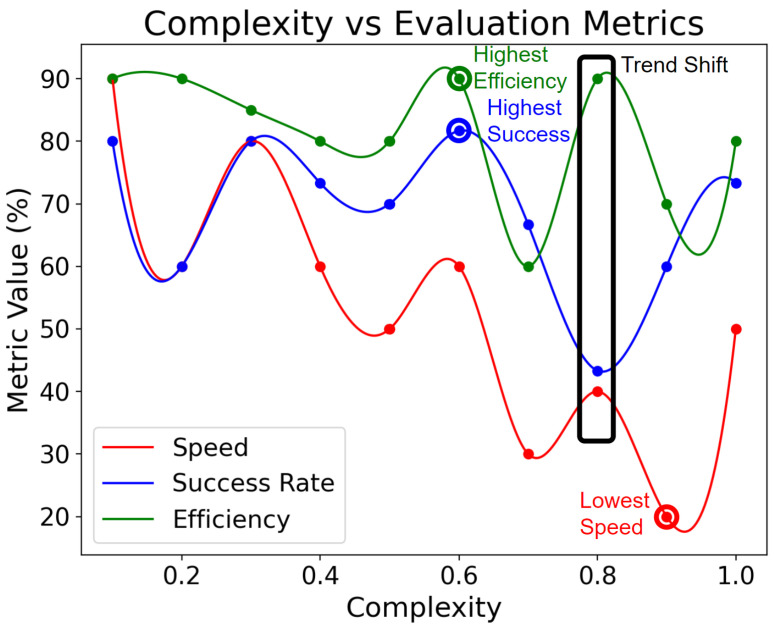
Message complexity vs Evaluation Metrics.

Figure 12 illustrates the relationship between message complexity and evaluation metrics with the temperature parameter set to 0.5. As complexity increases, the system’s performance decreases. The system achieves peak success rate and efficiency at a complexity level around 0.6, where the system can handle sophisticated user requests while maintaining high reliability. A notable shift occurs at a complexity of 0.8, where the success rate diverges from efficiency and speed. This divergence is attributed to the LLM correctly executing functions but struggling with conditional and sequential order. Such behaviors underscore the challenges in maintaining coherent task execution as complexity scales up, even when individual components are processed accurately. Beyond a complexity of 0.9, all three metrics exhibit an upward trend, indicating the model’s capability to handle highly complex tasks while maintaining high success rates, albeit with potential trade-offs in efficiency and speed. These findings highlight the delicate balance required in system design: while moderate complexity yields optimal overall performance, the system can adapt to highly complex inputs at the cost of reduced efficiency. This insight is crucial for tailoring the Vega system to different use cases, from simple smart home controls to complex industrial applications, where the balance between task complexity and system performance may vary based on specific requirements and operational contexts.

**Figure 13:**
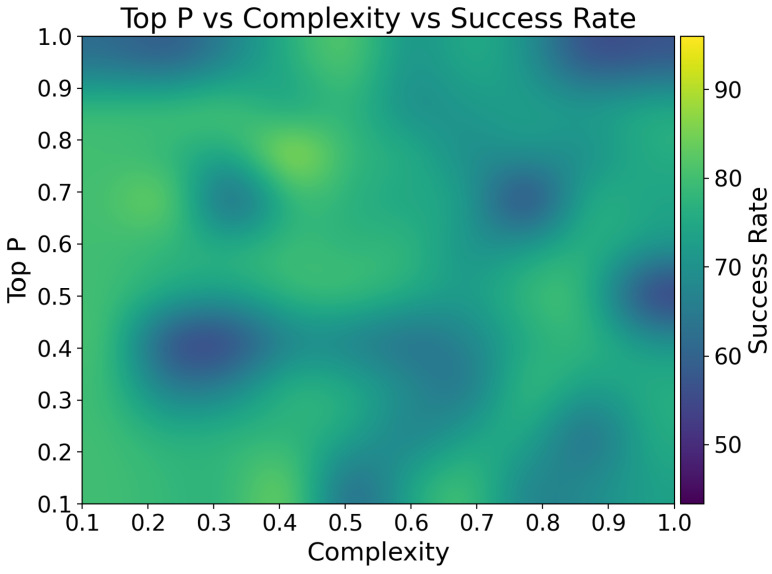
Heatmap for success rate against message complexity and Top P of the LLM.

Figure 13 provides additional insights into the relationship between message complexity and Top P parameter optimization in LLM-driven IoT control systems. Top P is an LLM parameter that limits token selection to the most probable choices that sum to a specified probability threshold [56]. The heatmap reveals distinct performance zones that inform optimal parameter selection strategies for different operational scenarios.

The analysis reveals that high success rates (85–90%) occur primarily in two distinct regions: low complexity (0.1–0.3) with mid-to-high Top *p* values (0.6–0.9), and moderate complexity (0.4–0.6) with mid-to-high Top *p* values (0.7–0.9). This pattern indicates that for simple IoT control tasks, a moderate degree of token diversity (Top P 0.6–0.9) provides sufficient flexibility for natural language interpretation without introducing excessive variability that could lead to misinterpretation.

For moderate-complexity tasks, the optimal zone shifts toward higher Top *p* values (0.7–0.9), suggesting that more complex instructions benefit from increased token diversity to explore various interpretation pathways. This finding is consistent with the observations of Singh et al. [19], who noted that programmatic prompts with well-defined action specifications lead to more successful plan generation in situated robot environments.

Notably, the heatmap shows reduced performance in high-complexity scenarios (0.8–1.0) regardless of Top P settings, indicating that extremely complex instructions challenge the system’s interpretation capabilities. However, within this high-complexity region, moderate Top *p* values (0.4–0.7) tend to perform better than extreme values, suggesting that a balanced approach to token selection diversity is crucial for handling complex multi-step IoT control sequences.

The visualization also reveals a performance cliff at very low Top *p* values (0.1–0.3) across all complexity levels, indicating that overly restrictive token selection limits the system’s ability to interpret natural language variations and colloquialisms commonly used in IoT control scenarios. This insight is particularly valuable for deployment in diverse user environments where linguistic patterns may vary significantly.

The primary LLM used for the evaluation in this project was GPT-3.5, developed by OpenAI. Although GPT-4 was not used in our main experiments, we anticipate that leveraging it could lead to a significant improvement in performance, given its larger training dataset and greater capacity. This expectation aligns with the findings of Wu et al. [22], who reported improved results in personalized robot assistance tasks when utilizing more advanced LLMs. Benchmarking both models in addition to other models can be achieved in future research.

These findings have significant implications for LLM-driven IoT control systems. The heatmap provides valuable guidance for understanding optimal configurations across different IoT scenarios, contributing to the design of more robust and intuitive LLM-powered interfaces. By carefully tuning parameters such as temperature, Top P, and task complexity, it is possible to achieve higher success rates and more reliable system performance.

The results also highlight the importance of considering the trade-offs between exploration (controlled by temperature and Top P) and task difficulty (complexity) in IoT control scenarios. This aligns with the observations of Vemprala et al. [18], who emphasized the need for balancing exploration and exploitation in robotics applications using LLMs.

Moreover, the non-uniform distribution of success rates across the parameter space underscores the need for adaptive parameter selection strategies in real-world IoT deployments. This is particularly relevant in dynamic environments where task complexity may vary, as noted by Singh et al. [19] in their work on integrating action knowledge and LLMs for task planning in open worlds.

The observed performance improvements with GPT-4 suggest that future advancements in LLM architectures and training methodologies may lead to even more capable IoT control systems. This potential for improvement is consistent with the varied capabilities of LLMs across different tasks, as highlighted by Kumar [1] in their comprehensive survey on the evaluation of LLMs across multiple domains and reasoning types.

However, it is important to note that while LLMs show promise in IoT control applications, they also present challenges related to reliability, interpretability, and security. As pointed out by Sarzaeim et al. [29] in their work on LLM-assisted smart policing systems, careful consideration must be given to the ethical implications and potential biases of LLM-driven decision-making in critical systems.

The experimental results provide valuable insights into the performance characteristics of LLM-powered IoT control systems. By understanding the relationships between input parameters and success rates, developers can optimize these systems for improved reliability and effectiveness across a wide range of IoT applications.

### 4.7. Error Analysis with Concrete Examples

**Figure 14:**
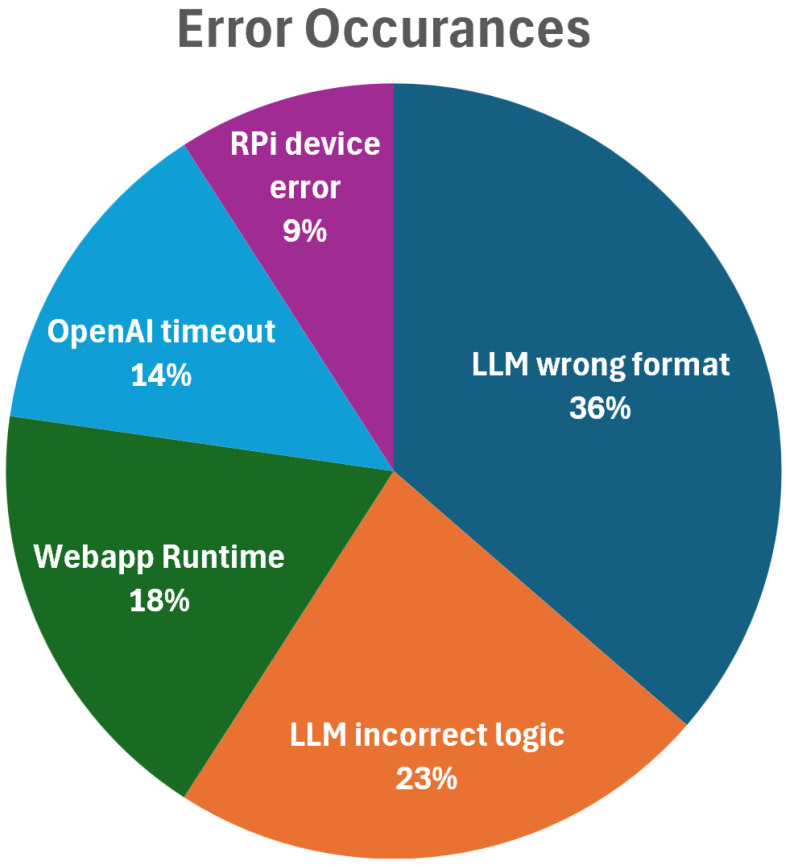
What types of errors occurred throughout testing.

Figure 14, titled “Error Occurrences,” shows a pie chart of errors in the Vega platform. LLM-related issues dominate, with wrong format and incorrect logic making up 59% of errors, highlighting the challenges of LLM integration and the need for better prompt engineering or parsing. The error distribution reflects the platform’s complexity, where OpenAI timeout errors (14%) suggest potential API-related performance bottlenecks, and web app runtime errors (18%) point to user interface stability issues. The lower rate of RPi device errors (9%) indicates reliable hardware, with most challenges residing in software and AI integration.

To provide a clearer understanding of system limitations and guide future improvements, a detailed analysis of error types with concrete examples is presented below:

Wrong Format Errors (36% of total errors): These occur when the LLM generates responses that do not conform to the expected function call structure. For example, when asked to “turn on the LED and check temperature,” the system might generate {“action”: “led_control”, “parameters”: “on”} instead of the required format {“function”: “set_led”, “parameters”: {“state”: “on”}}. These errors typically result from inconsistent prompt engineering or insufficient examples in the training context.

Incorrect Logic Errors (23% of total errors): These represent failures in conditional logic interpretation. A concrete example involves the command “If temperature is above 25 °C, turn on fan, otherwise turn on LED.” The system might execute both actions simultaneously or fail to evaluate the condition properly. For instance, it might call set_fan(“on”) and set_led(“on”) regardless of temperature, indicating challenges in parsing conditional statements.

Web App Runtime Errors (18% of total errors): These encompass user interface failures and communication breakdowns. Examples include WebSocket connection timeouts during prolonged interactions, memory leaks from accumulated chat history, and state synchronization issues between the frontend and backend. A typical scenario involves users losing connection mid-conversation, resulting in incomplete command execution and user confusion.

OpenAI Timeout Errors (14% of total errors): These occur when API calls exceed response time limits, particularly during peak usage periods or with complex queries. For example, a command like “analyze the last hour of sensor data and create a comprehensive report with recommendations” might time out due to processing complexity, leaving users without feedback or partial results.

RPi Device Errors (9% of total errors): Hardware-related failures include sensor malfunctions, GPIO communication errors, and peripheral device unresponsiveness. Specific examples include temperature sensor drift leading to erroneous readings (e.g., reporting −40 °C in room temperature), servo motor jamming during positioning commands, and relay contact degradation causing inconsistent switching behavior.

Each error type has distinct impact patterns on user experience and system reliability. Wrong format and incorrect logic errors primarily affect task success rates and require user intervention or retry attempts. Web app runtime errors disrupt user workflow and may result in data loss or system state inconsistency. OpenAI timeout errors create uncertainty about command execution status and may lead to duplicate actions. RPi device errors compromise system reliability and may require manual intervention or hardware maintenance.

These concrete examples inform targeted improvement strategies: enhanced prompt engineering for format consistency, improved logical parsing algorithms, robust error handling and retry mechanisms, timeout management and progress indication, and predictive maintenance for hardware components.

**Figure 15:**
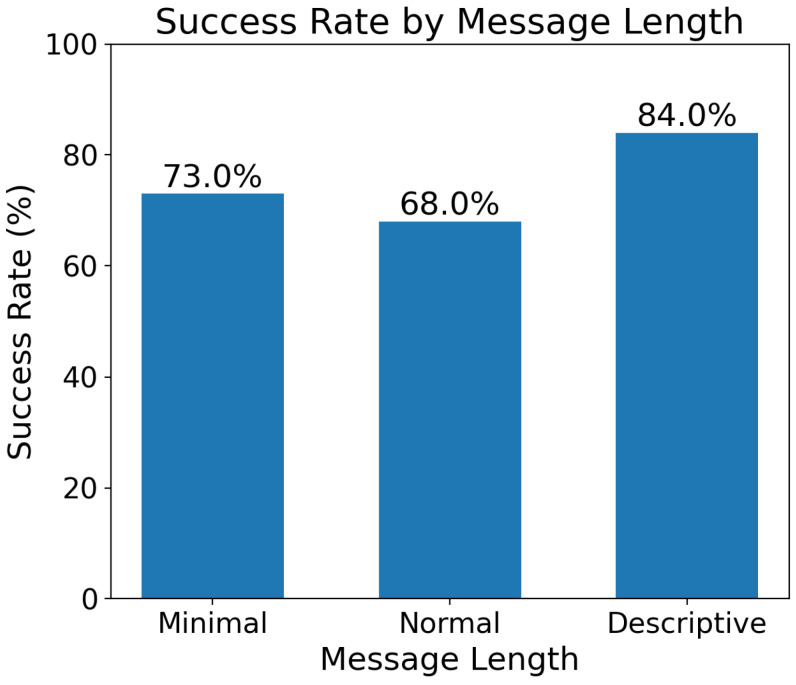
Success rate of different tones of the same message.

Figure 15 illustrates the success rate of message interpretation by the system across three message length categories: minimal (73.0%), normal (68.0%), and descriptive (84.0%). Notably, the descriptive messages achieve the highest success rate, indicating that more detailed inputs significantly improve performance. Interestingly, minimal messages outperform normal ones, recommending that concise commands may reduce ambiguity. The lower success rate for normal messages implies a potential trade-off between brevity and specificity, emphasizing that either highly detailed or very concise communication may be optimal. While the normal category exhibits a comparatively lower success rate, it still maintains a commendable performance. This suggests that even in less optimal conditions, the system demonstrates robust functionality. Furthermore, the potential integration of more advanced language models, such as GPT-4, could significantly enhance these success rates across all categories, potentially pushing the system’s overall performance to new heights.

The LLM, while highly capable in general language understanding, may lack specialized knowledge in IoT hardware control. This could lead to misinterpretations or errors when dealing with intricate or domain-specific instructions. While utilizing OpenAI’s API incurs ongoing costs, it eliminates the need for extensive local computational resources and the time-intensive process of model training. However, for scenarios requiring extremely high accuracy or dealing with highly specialized IoT vocabularies, future iterations of the system might benefit from fine-tuning or developing domain-specific models.

In summary, our experimental results reveal several key insights. The system demonstrates a high level of proficiency in interpreting and executing complex commands, particularly excelling with descriptive inputs, achieving an impressive 84% success rate. We observed that performance fluctuates based on message complexity and LLM parameters, with optimal results occurring at moderate complexity levels (0.4–0.6) and higher temperature settings (0.7–1.0). Notably, different IoT functions exhibit varying degrees of success and execution speeds, with text-based operations such as LCD control performing exceptionally well.

## 5. Conclusions

### 5.1. Limitations

The current implementation of our system, while innovative, faces several limitations that warrant acknowledgment. Our choice to utilize GPT-3.5 instead of GPT-4, driven by cost considerations at the time of the study, may have constrained the system’s overall performance and capability. Our analysis uncovered that the majority of observed errors (59%) stem from LLM-related issues, including incorrect formatting and logic, which clearly indicates areas for future improvement and refinement of the system; however, utilizing GPT-4 could help mitigate those issues. A significant concern arises from the reliance on OpenAI’s cloud-based service, which introduces potential data privacy issues as user interactions are processed externally. Despite extensive testing across various scenarios, the inherent unpredictability of LLMs remains a challenge, with the possibility of misinterpreting user commands or producing inconsistent responses. The prohibitive cost of fine-tuning at scale presents a barrier to improving the system’s accuracy and reliability. Moreover, the current architecture lacks support for real-time feedback, limiting the fluidity of user interactions. The system’s dependency on specific hardware (RPi) and software (Python-based server) configurations may restrict its applicability in diverse IoT environments.

### 5.2. Future Work

Future work aims to address these limitations and expand the system’s functionality and applicability. A primary objective is the implementation of real-time feedback mechanisms, enabling live interactions between the LLM, web application, and user, thus enhancing the responsiveness and intuitiveness of the interface. Developing a framework for repeatable logic execution would allow complex commands to run periodically on the IoT device without constant LLM oversight, improving efficiency and reducing computational load. Expanding support to C/C++ based IoT platforms such as STM32 and Arduino would significantly broaden the system’s compatibility with diverse hardware ecosystems. The integration of an MQTT server alongside the existing Flask server could enhance IoT interoperability, allowing for more flexible and real-time device communication. Exploring options for local LLM hosting and investigating alternative, potentially open-source LLM solutions could mitigate privacy concerns and potentially reduce operational costs. Additionally, future research could focus on developing more sophisticated natural language understanding capabilities, enabling the system to handle increasingly complex and context-dependent user queries. Lastly, future work should focus on developing adaptive parameter selection strategies, improving LLM performance on high-complexity tasks, and addressing the ethical and security considerations associated with LLM deployment in IoT environments. These enhancements would collectively contribute to a more versatile, secure, and user-friendly IoT interface, paving the way for broader adoption in smart homes, industrial settings, and educational environments.

### 5.3. Critical Analysis and Real-World Deployment Challenges

While our system demonstrates promising capabilities in controlled environments, a critical examination reveals significant challenges that must be addressed for real-world IoT deployment. The fundamental reliance on cloud-based LLM services introduces several operational vulnerabilities that extend beyond privacy concerns. Network connectivity dependencies present a critical failure point in IoT environments where internet access may be intermittent or unreliable, particularly in industrial, remote, or disaster-recovery scenarios. The system’s current architecture would be entirely non-functional during network outages, highlighting the need for hybrid approaches that incorporate local processing capabilities.

The scalability challenges become particularly acute when considering enterprise-level deployments. Our current implementation, while effective for single-device control, faces exponential complexity increases when managing multiple IoT devices simultaneously. The token consumption costs associated with cloud-based LLM services could become prohibitive in large-scale deployments, where thousands of devices generate continuous interaction streams. Furthermore, the latency introduced by cloud processing may be incompatible with time-critical IoT applications such as industrial automation, autonomous vehicles, or emergency response systems, where millisecond-level response times are essential.

Security considerations extend beyond data privacy to encompass device integrity and system reliability. The interpretation of natural language commands by LLMs introduces potential attack vectors through prompt injection or adversarial inputs that could compromise device functionality or safety. In safety-critical IoT environments such as healthcare monitoring or industrial control systems, the probabilistic nature of LLM responses poses unacceptable risks where deterministic behavior is mandatory. The lack of formal verification mechanisms for LLM-generated commands represents a significant limitation for applications requiring regulatory compliance or safety certification.

The heterogeneity of IoT ecosystems presents additional deployment challenges not fully addressed in our current implementation. Real-world IoT networks typically comprise devices with varying computational capabilities, communication protocols, and power constraints. Our system’s current dependency on RPi-class hardware and Python-based implementations may not translate effectively to resource-constrained devices that are common in IoT deployments, such as microcontroller-based sensors with limited memory and processing power.

Operational maintenance and system evolution present further complications in real-world scenarios. The rapid evolution of LLM technologies and potential deprecation of cloud services could render deployed systems obsolete or non-functional. The lack of version control and rollback mechanisms for LLM behavior changes poses risks for systems requiring stable, predictable operation over extended periods. Additionally, the specialized knowledge required for system maintenance and troubleshooting may negate some of the accessibility benefits the system aims to provide, particularly in environments where technical expertise is limited.

Despite these challenges, our research provides valuable insights into the potential and limitations of LLM-driven IoT control. The identification of these real-world deployment barriers serves as a foundation for future research directions and highlights the importance of developing robust, locally deployable solutions that can operate reliably in diverse and challenging IoT environments.

### 5.4. Conclusions

This project successfully developed and evaluated a novel system that integrates NLP with IoT infrastructure through LLM-mediated control mechanisms. The system consists of a web application interfacing with an LLM to interpret user commands, which are executed on an RPi controlling a physical circuit. It features a modular and scalable architecture with comprehensively documented components, including capabilities such as multimodal input through image recognition, complex task interpretation, an accessible and user-friendly chat application supporting various data visualizations, including plots and flow charts, a modular server on the RPi, and compatibility with a wide range of circuit devices.

Our evaluation results demonstrate the system’s effectiveness in handling complex tasks with high success rates, particularly when appropriate LLM parameter settings are employed. Case studies showcase real-world applicability scenarios such as machinery monitoring and drone delivery systems. The system’s modular design facilitates the easy integration of different circuit components and employs intelligent agents for enhanced robustness, establishing a comprehensive and user-friendly solution for IoT and embedded systems development.

However, our critical analysis reveals significant limitations that temper the immediate applicability of such systems in production IoT environments. The dependencies on cloud-based services, network connectivity requirements, scalability constraints, security vulnerabilities, and operational challenges represent substantial barriers to widespread adoption. These findings underscore the complexity of translating research prototypes into robust, deployable IoT solutions and highlight the need for continued research into hybrid architectures that balance the power of LLMs with the reliability requirements of real-world IoT applications.

The true innovation lies in the novel combination of components and the intelligent layer that bridges them. By leveraging LLMs for intuitive IoT control, our system enhances human–machine interaction paradigms and demonstrates the potential for making IoT technologies more accessible to non-technical users. The system’s emphasis on modularity, security awareness, and scalability considerations provides a foundation for accommodating new circuit components and visualizations while addressing some of the ethical and operational concerns inherent in LLM-driven systems.

The societal implications of this research extend beyond technical achievements to encompass improved automation efficiency, enhanced technological literacy, the promotion of responsible AI practices, and customization capabilities that address diverse user needs and ethical concerns. While the current implementation faces significant real-world deployment challenges, it establishes important precedents for human-centric IoT interfaces and provides a critical foundation for future research into more robust, locally deployable solutions.

Overall, this project marks a significant step toward understanding both the potential and limitations of integrating natural language processing with IoT infrastructures. By providing an honest assessment of current capabilities alongside a critical examination of deployment challenges, this research contributes to the broader discourse on responsible AI implementation in IoT environments and lays the groundwork for future advancements in intelligent automation and human–machine interaction systems that can operate reliably in diverse real-world contexts.

## Figures and Tables

**Table 1:** Comparative analysis of natural language IoT control systems.

System	NL Capability	IoT Integration	Accessibility	Deployment	Key Limitations
Traditional GUI	None	Native	Low	Simple	Requires technical knowledge
Voice Assistants (Alexa/Google)	Limited	Skill-based	Medium	Medium	Predefined commands, limited context
Rule-based Systems (IFTTT)	None	Good	Low	Medium	No natural language, rigid logic
Chat2VIS [17]	High	Limited	Medium	Complex	Visualization-focused, not IoT control
CASIT [28]	Medium	Good	Medium	Complex	Limited user interaction paradigms
ProgPrompt [19]	High	Robotics only	Medium	Complex	Robotics-specific, not general IoT
PaLM-E [20]	High	Limited	Low	Very Complex	Requires extensive training, resource-intensive
Vega (This Work)	High	Native	High	Medium	Requires internet connectivity

**Table 2:** Physical devices defined on the control server, which are then utilized by the LLM to interact with the circuit.

Symbol	Pin Type	Description
ULTS	Input	Ultrasonic distance sensor in cm
CAM	Input	Camera device for image acquisition
GPS	Input	GPS device for longitude and latitude coordinates
TMP	Input	Temperature sensor in degrees celsius
FAN	Output	12V fan controlled through digital GPIO in relay
LCD	Output	LCD for displaying text data
SRV	Output	Servo motor rotates to given set of angles
LED1	Output	Yellow LED light
LED2	Output	Red LED light
LED3	Output	Blue LED light

**Table 3:** Defined functions on the control server, called by the LLM based on user input, executed on the RPi and processed on the web app.

Function	Description	Use Case
set_led	Toggles specific LED	“Turn on yellow LED”
set_fan	Toggles fan on or off	“Turn on the fan”
get_recorded_sensor_data	Gets interval sensordata from database	“Plot me the distance data in last 30 s”
get_raspberry_stats	Gets CPU, RAM, disk of RPi	“What is the current disk usage”
capture_image	Capture and uploadimage to the cloud	“Capture an image, does it contain a pen?”
get_connected_devices	Fetches data of connected devices	“What is the current humidity and temperature”
get_location_	Gets the current location from GPS	“From the location are we currently in Leeds?”
set_servo_angles	Turn servo to certain angle	“Turn the servo to 10 then 180 degrees”

## Data Availability

The data presented in this study are available on request from the corresponding author due to privacy.
